# Feasibility of myocardial perfusion assessment with contrast echocardiography: can it improve recognition of significant coronary artery disease in the ICU?

**DOI:** 10.1186/s13054-019-2519-1

**Published:** 2019-07-17

**Authors:** Sam Orde, Michel Slama, Faraz Pathan, Stephen Huang, Anthony Mclean

**Affiliations:** 1 Intensive Care Unit, Nepean Hospital, Sydney, 2750 Australia; 2 Medical ICU, Amiens University Hospital, Amiens, France; 3 Cardiology Department, Nepean Hospital, Sydney, 2750 Australia; 4 Intensive Care Unit, Nepean Hospital, Kingswood, Sydney, NSW 2749 Australia

**Keywords:** Critically ill, Contrast, Echocardiography, Perfusion

## Abstract

**Background:**

Diagnosis of significant coronary artery disease (CAD) and acute coronary artery occlusion in ICU can be difficult, and an inappropriate intervention is potentially harmful. Myocardial contrast perfusion echo (MCPE) examines ultrasound contrast intensity replenishment curves in individual myocardial segments measuring peak contrast intensity and slope of return as an index of myocardial blood flow (units = intensity of ultrasound per second [dB/s]). MCPE could possibly serve as a triage tool to invasive angiography by estimating blood flow in the myocardium. We sought to assess feasibility in the critically ill and if MCPE could add incremental value to the clinical acumen in predicting significant CAD.

**Methods:**

This is a single-centre, prospective, observational study. Inclusion criteria were as follows: adult ICU patients with troponin I > 50 ng/L and cardiology referral being made for consideration of inpatient angiography. Exclusion criteria were as follows: poor echo windows (2 patients), known ischaemic heart disease, and contrast contraindications. Seven cardiologists and 6 intensivists blinded to outcome assessed medical history, ECG, troponin, and 2D echo images to estimate likelihood of significant CAD needing intervention (clinical acumen). Clinical acumen, quantitative MCPE, and subjective (visual) MCPE were assessed to predict significant CAD.

**Results:**

Forty patients underwent MCPE analysis, 6 (15%) had significant CAD, and median 11 of 16 segments (IQR 8–13) could be imaged (68.8% [IQR 50–81]). No adverse events occurred. A significant difference was found in overall MCPE blood flow estimation between those diagnosed with significant CAD and those without (3.3 vs 2.4 dB/s, *p* = 0.050). A MCPE value of 2.8 dB/s had 67% sensitivity and 88% specificity in detecting significant CAD. Clinical acumen showed no association in prediction of CAD (OR 0.6, *p* = 0.09); however, if quantitative or visual MCPE analysis was included, a significant association occurred (OR 17.1, *p* = 0.01; OR 23.0, *p* = 0.01 respectively).

**Conclusions:**

MCPE is feasible in the critically ill and shows better association with predicting significant CAD vs clinical acumen alone. MCPE adds incremental value to initial assessment of the presence of significant CAD which may help guide those who require urgent angiography.

## Background

Significant coronary artery disease (CAD) and acute coronary artery occlusion can be challenging to diagnose in the critically ill [1]. Accurate diagnosis is important as unnecessary angiographic intervention or anti-thrombotic therapy can be harmful, particularly in those with multi-organ dysfunction. Medical history, examination, ECG analysis, echo, and other investigations are all important for diagnosis but can lack precision in the ICU. Troponin levels, in particular, can be elevated in critically ill patients reflecting myocardial damage, but this may occur through several mechanisms other than significant CAD and/or acute atherosclerotic plaque disruption [1].

**Fig. 1:**
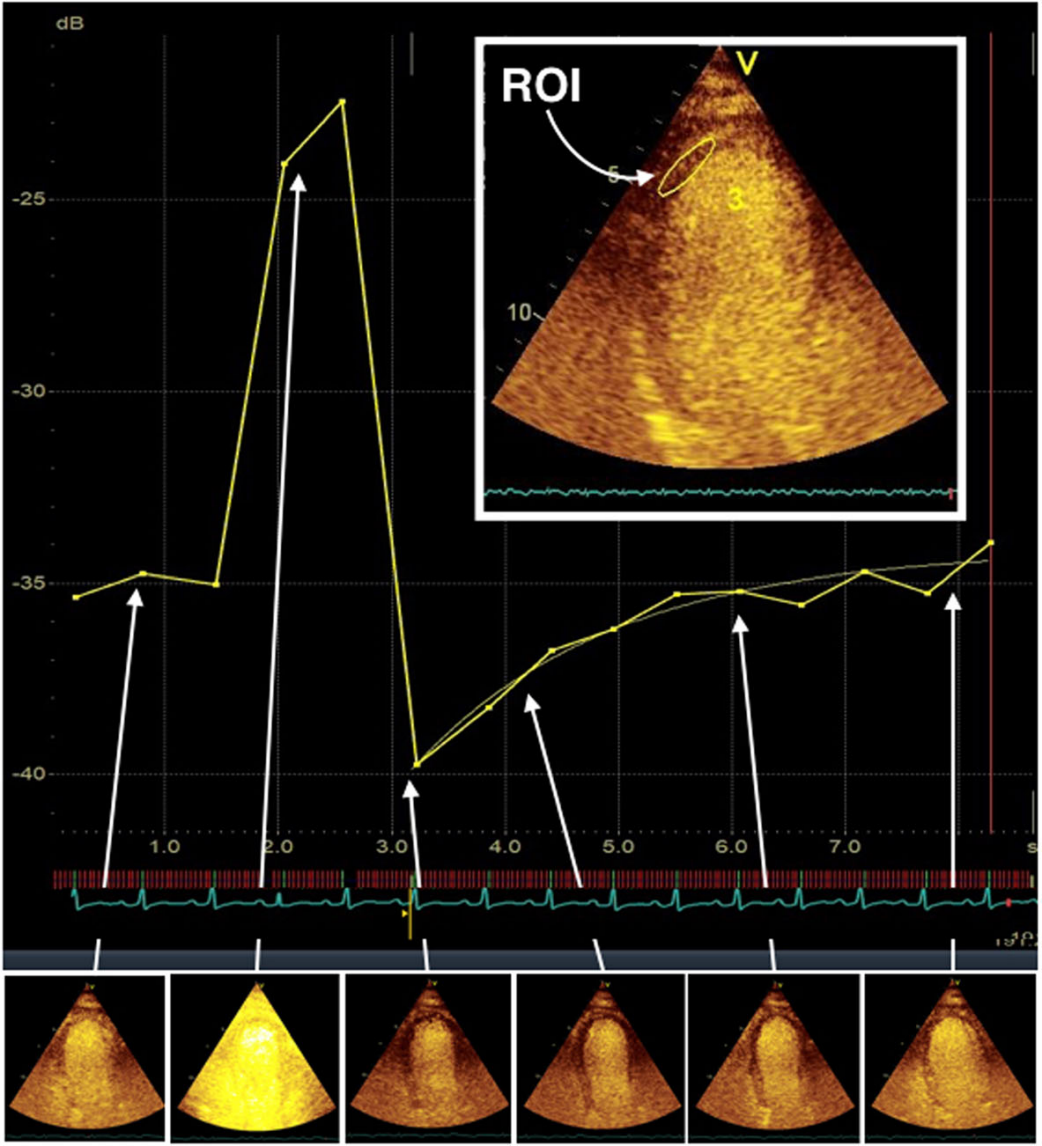
Myocardial contrast perfusion echocardiography (MCPE): quantitative analysis. Echo contrast microbubbles are small enough to pass through the microcirculation, and this feature can be used to estimate myocardial blood flow. A region of interest (ROI) is defined in a myocardial segment and the signal intensity at the plateau and the rate of change at each end-diastolic frame are analysed to estimate myocardial blood flow

Myocardial perfusion assessment with echo contrast (known as myocardial contrast perfusion echocardiography [MCPE]) is a technique used predominantly in stress echo studies for simultaneous assessment of myocardial perfusion and regional wall motion abnormality. It has been shown to improve the detection of CAD, in a safe manner and can have prognostic value over regional wall motion detection [2, 3]. Echo contrast agents (e.g. Definity) are microbubbles of inert gas surrounded by a stabilizing shell (e.g. perflutren carbon) typically 1–8 μm in diameter. These bubbles are injected into the venous system and are small enough to pass through the pulmonary microvasculature to then pass into the systemic circulation. This allows for the labelled use of this agent for left ventricle (LV) opacification to improve detection of thrombus, regional wall motion abnormalities, accurate ejection fraction estimation, etc. [4]. Low-intensity ultrasound waves are needed when imaging with echo contrast to prevent destruction of the fragile bubbles. However, this feature can be used to an advantage by applying a burst of high-intensity ultrasound for a short period of time; bubbles are destroyed; and through analysing the ‘replenishment’ rate, as contrast bubbles trickle back in to the myocardial circulation, perfusion can then be assessed [5] (see Fig. 1).

Echo contrast agents have been shown to be safe in the critically ill for LV opacification [6]. This study sought to assess the feasibility of MCPE in ICU patients with raised troponin levels being referred for inpatient coronary angiography for suspected acute coronary artery occlusion. Furthermore, we pursued if quantitative or subjective (i.e. visual) assessment with MCPE could aid in the diagnosis of acute coronary artery occlusion. It was hypothesized that MCPE would be feasible, improve recognition, and add incremental value to clinical acumen.

## Methods

### Study design

We performed a prospective, observational study in the ICU at Nepean Hospital, Sydney, Australia, between May 2014 and January 2017 on non-consecutive patients (S.O. is the sole MCPE operator in the unit, hence dependent on availability). All patients or authorized representatives (next of kin) gave written consent to be involved in the study which was approved by the Nepean Blue Mountains Local Health District human research and ethics committee (study 15/17-LNR/15/NEPEAN/37). Inclusion criteria were as follows: adult (> 18 years), raised (high sensitivity) troponin I levels (greater than 50 ng/L), acute coronary artery occlusion being considered, and a request made for consideration of coronary angiography. Patients were excluded if they had urgent angiography already performed due to STEMI criteria being met, were unable to have apical echo imaging performed (2 patients), past medical history of ischaemic heart disease, contraindications to echo contrast (allergies, known significant intracardiac shunts, severe pulmonary hypertension), significant valvular abnormalities, pregnant, and study refusal.

Six intensive care specialists with a high level of echo experience (i.e. DDU qualification or equivalent) as well as seven cardiologists were invited to review all relevant patient data and echo imaging to provide an estimate, based on clinical acumen, of the likelihood of the presence of significant CAD needing inpatient intervention on a Likert scale. Data included admission history, ECG, serum troponin levels, and past medical history (particularly including history of hypertension, diabetes, smoking, family history). In addition, time of admission, imaging and troponin, APACHE III, SOFA score, and haemodynamic data were recorded. The presence of significant CAD was assessed by coronary angiography, nuclear imaging, MRI, CTCA, or normal repeat echocardiography shortly after initial imaging in patients with stress-induced cardiomyopathy diagnosis.

### Echocardiography and myocardial contrast perfusion echocardiography (MCPE) imaging

A full comprehensive echo was performed initially by S.O. or trained sonographers with either a Vivid 9 or Vivid I echo machine (General Electric, Boston, MA, USA). The studies and analysis were performed in accordance with the leading echo organization guidelines [7, 8] to obtain LV size, ejection fraction, and regional wall motion abnormalities to gain a wall motion severity index score: average of 16 segment model score based on normal thickening = 1, hypokinesis = 2, akinesis = 3, and dyskinesis = 4. In addition, speckle tracking echocardiography (STE) analysis was also performed to determine global longitudinal strain. STE analysis was completed in manner as previously described [9, 10] by S.O. (who has performed over 1000 analyses) in accordance with a consensus document from leading organizations [11]. S.O. then performed the MCPE examination with a Vivid 9 echo machine with a M3S matrix array transducer, at the earliest time frame possible from the inclusion criteria being met.

The contrast agent Definity® was used for MCPE analysis. The microspheres have a mean bubble size of 1–10 μm enabling passage through the pulmonary vasculature [4]. Definity was drawn up into a 20-ml syringe with normal saline and injected in one to two ml increments to enable a homogenous contrast enhancement in the myocardium with no attenuation. Once imaging was optimized, a flash of higher-intensity ultrasound (MI ~ 1.0) for 30 frames (to cover at least one systolic period) timed to coincide with systole on the ECG, was manually triggered. Images were recorded for two to five beats prior to the flash and 8–15 beats after the flash to adequately assess for replenishment.

### MCPE image analysis

**Fig. 2:**
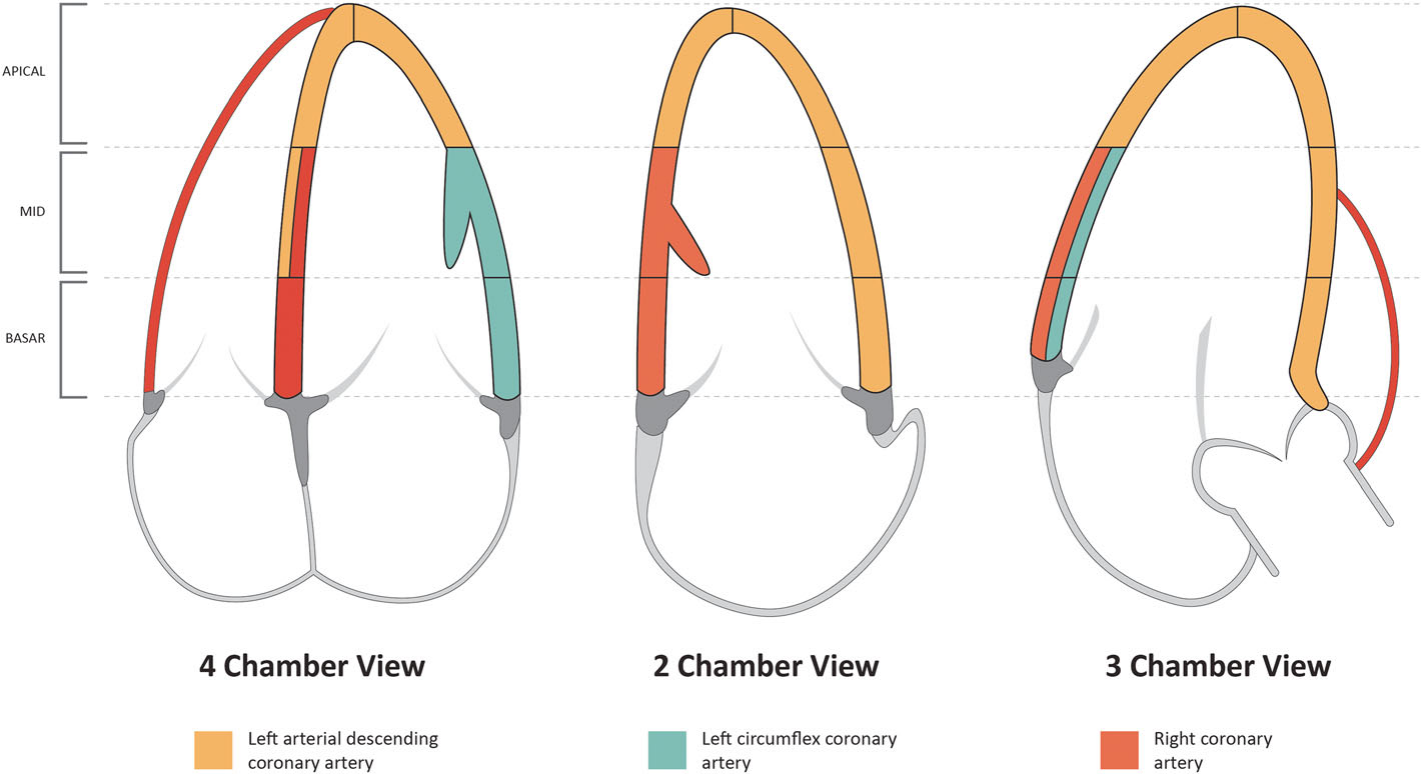
Segmental coronary artery territory vascular supply used for feasibility assessment of myocardial contrast perfusion echocardiography (MCPE) and 2D echo analysis

Images were transferred to an EchoPACS reporting station (General Electric, Boston, MA, USA) for off-line quantitative analysis for each segment that was able to be visualized. Analysis was attempted to be performed in a blinded fashion to achieve outcome results in a manner as previously described in other studies [12, 13]. Subjective (visual) analysis was performed with a simple scoring system: 0 normal, 1 contrast perfusion deficit. Quantitative analysis was performed by measuring ultrasound signal intensity in the ‘region of interest’ (ROI) at each myocardial segment following a standard 16-cardiac segment model (see Fig. 2). The ROI size was optimized to include as much of the myocardial segment as possible while avoiding the low-intensity signals from the pericardium or high-intensity signals from the LV cavity. The first end-systolic frame after the ‘flash’ was considered *t*_0_, and signal intensity (SI) was calculated for each ROI at each end-systolic frame and plotted against time and fitted to the exponential function: *y*(*t*) = *A*(1 − e^−*β*(*t* − *t*0)^) + *C*. *y* is the SI in the ROI at the end-systolic frame, *A* is the plateau SI corresponding to myocardial blood volume, *β* is the exponential decay function (decay constant) representing the rate of SI rise, and *C* is the intercept at the origin reflecting the background intensity level. *A* × *β* provides an estimate of the initial rate of contrast replenishment, and this provides a surrogate of myocardial blood flow [14].

Feasibility of both subjective and quantitative analysis was assessed at segmental and coronary artery territory distribution (see Fig. 2) as well as the apex vs mid vs basal levels. Segments were excluded from analysis if the myocardium vasculature lacked opacification on visual and quantitative analysis. Coronary artery territories were considered ischaemic if a third (or more) of the segments in that territory had impaired contrast filling. The left anterior descending (LAD) and right coronary artery (RCA) territories were considered unable to be assessed if two segments were unable to be examined. The left circumflex coronary artery (LCx) territory was considered unable to be assessed if one segment could not be examined.

### Statistical analysis

Due to the exploratory nature of the study, a sample size was not formally calculated and a number of 40 patients were felt suitable to make an initial assessment and guide future research. Statistical analysis was performed with JMP Pro version 13 (SAS Institute Inc., Cary, NC, USA). Continuous variables are expressed as mean ± standard deviation (SD) if normally distributed, and as median with interquartile range (IQR) if not normally distributed. Normality was assessed using the Shapiro-Wilk test. Feasibility was defined as the number of segments where analysis was possible compared to total segments from all included patients. A between-group comparison for continuous data was performed by the Student *t* test and non-parametric or non-normally distributed data by the Wilcoxon signed-rank/Kruskal-Wallis (rank sum) test. Categorical data was compared by Pearson’s chi-squared analysis or Fisher’s exact test. All probability values are two-sided, and *p* values < 0.05 are considered statistically significant. Logistic regression was used to assess the association between the presence of acute coronary artery occlusion and clinical acumen and/or subjective or visual MCPE analysis. Receiver operating curves were analysed to determine optimal MCPE values for the presence or absence of acute coronary artery occlusion. Inter-rater variability was performed on a random 15% of patients for myocardial blood flow estimation by MCPE and assessed by absolute difference and expressed as a percentage of their mean.

## Results

Forty patients, 70% female, mean age 59.8 (± 17), were included in the study of which six were confirmed to have significant CAD (15%), all by coronary angiography: two patients with right coronary artery ischaemia, one patient with the left circumflex coronary artery occluded, and three with both the left anterior descending and left circumflex coronary artery occluded. No adverse reactions to echocardiography contrast were seen. Angiography was performed in 22 patients (55%). Normal non-invasive studies led to the decision not to proceed with angiography in the remaining 18 patients: normal follow-up echo in 11 (28%), normal CT coronary angiogram in 3 (8%), and normal MRI perfusion study in four (10%). Demographic, clinical and initial investigation data is presented in Table 1. No major baseline differences were seen between patients with no significant CAD found vs those with the disease, except diabetes which was more prevalent in the significant CAD group (*p* = 0.001). Patients were critically ill as demonstrated by a mean SOFA score of seven (equating to a 15–25% risk of ICU death [15]) and a mean APACHE III score of 73 (estimated risk of hospital death approximately 25–35% [16]), with 43% requiring catecholamine support and 53% mechanically ventilated. Risk factors for CAD were commonly seen in both groups (particularly hypertension and smoking, seen in 40% and 35% respectively).

**Table 1:** Patient demographics, clinical parameters, and relevant investigation results

	Characteristic		Overall	No coronary artery disease	Significant coronary artery disease	*p* valve
Demographics	Number (*n*, %)		40	34 (85%)	6 (15%)	–
	Female (%)		28 (70%)	25 (74%)	3 (50%)	0.25
	Age (years)		59.8 (± 17)	58.1 (± 17)	69.4 (± 18)	0.02
	Past medical history	Hypertension (%)	16 (40%)	13 (38%)	3 (50%)	0.67
		Diabetes (%)	9 (22%)	*4 (12%)*	*5 (83%)*	*0.001*
		Smoking (%)	14 (35%)	11 (32%)	3 (50%)	0.65
		Family history (%)	4 (10%)	3 (9%)	1 (17%)	0.55
Clinical parameters	Blood pressure	Systolic (mmHg)	117 (102–127)	114 (101–128)	122 (± 10)	0.9
		Diastolic (mmHg)	64 (± 13)	65 (± 14)	60 (± 13)	0.39
		Mean (mmHg)	79 (68–94)	83 (± 16)	80 (± 10)	0.57
	Sinus rhythm (*n*, %)		38 (95%)	32 (94%)	6 (100%)	1.0
	Heart rate (beats per min)		86 (18)	87 (± 19)	78 (± 8)	0.07
	Weight (kg)		80.4 (± 24)	79.9 (± 25)	83.7 (± 18)	0.7
	GCS		11 (3–15)	11 (3–15)	11 (5–15)	0.5
	Catecholamines required (*n*, %)		17 (43%)	16 (47%)	1 (17%)	0.2
	Dose (μg/kg/min)		15.1 (± 10)	15.1 (± 10)	15	–
	Mechanical ventilation (*n*, %)		21 (53%)	18 (53%)	3 (50%)	1.0
	PaO_2_ (mmHg)		78 (68–89)	75 (66–88)	88 (± 20)	0.5
	Platelets (ng/dL)		231 (± 102)	230 (± 103)	239 (± 106)	0.9
	Creatinine (ng/dL)		91 (61–170)	89 (61–171)	122 (± 64)	0.8
	Bilirubin (ng/dL)		6.5 (5–15)	8 (5–15)	5 (4.5–17)	0.3
	SOFA score		7 (5)	7 (5)	6 (5)	0.6
	APACHE III		73 (32)	72 (34)	82 (7)	0.3
Investigations	ECG	ST elevation, *n*(%)	5 (13%)	5 (15%)	0	–
		T wave inversion or flattening, *n*(%)	29 (73%)	24 (71%)	5 (83%)	1.0
		ST depression, *n*(%)	6 (15%)	4 (12%)	2 (33%)	0.21
	Biomarkers	Troponin I (ng/mL)	1987 (400–4384)	1943 (357–4182)	3016 (1255–8630)	0.28
	Echo	LV end diastolic diameter (mm)	49.0 (8)	48.6 (8)	50.7 (7)	0.54
		LV ejection fraction (%)	45.7 (15)	46.1 (15)	43.2 (16)	0.69
		Wall motion score index	2.0 (1.4–2.4)	2.0 (1.5–2.4)	1.8 (0.5)	0.95
		Global longitudinal strain (%)	− 9.2 (5)	− 9 (− 11 to − 6)	− 7 (− 16 to − 7)	0.9

The italicized data was simply meant to highlight the values which are statistically significant (ie: have p values <0.05)

No significant differences were seen between the groups in terms of investigations performed assessing for possible acute ischaemia, including ECG, troponin I blood tests, and echo. The most common ECG finding was T wave inversion or flattening (seen in 73%). Troponin I levels were severely raised in both groups (median 1943 [357–4182] vs 3016 [1255–8630] for those with no significant CAD vs those with significant CAD respectively). Echo was performed within median 8 h (3–22) from when troponin levels were taken. Echo data displayed predominantly a normal LV size: mean end diastolic diameter 49 mm (8); with mildly reduced LV systolic function measured by conventional ejection fraction: mean 45.7% (15); but with global longitudinal strain analysis by speckle tracking, more severe dysfunction was elucidated: mean − 9.2% (5). Substantial regional wall motion abnormalities were common (median wall motion score index 2 [1.4–1.8]).

### Feasibility analysis

Feasibility and values for each myocardial segment analysis technique are shown in Table 2. Quantitative analysis was estimated to be performed 24 h–3 weeks from time of echo. 2D segmental thickening analysis showed the greatest feasibility (in 90–100% of patients) and subjective MCPE analysis the least (20–53% of patients). Despite both 2D segmental thickening assessment and longitudinal strain analysis by STE being feasible in the majority of segments assessed, there were no significant differences seen in wall motion score index or longitudinal strain analysis values between groups with significant CAD and those with no CAD diagnosed. However, in both subjective and quantitative MCPE assessment, significant differences were seen (except in the right coronary artery territory in quantitative assessment). Quantitative MCPE analysis had better feasibility than subjective MCPE assessment. The LAD territory was the most feasible to be assessed (65% by quantitative MCPE and 53% by subjective MCPE analysis) and the LCx the least (20% by quantitative MCPE and 33% by subjective MCPE analysis). The feasibility of MCPE subjective and qualitative analysis was greatest at the apical level (89% and 90% respectively). The mid-level was easier to analyse (66% and 68% respectively) than the basal level (39% and 48% respectively).

**Table 2:** Feasibility and results (overall and for individual coronary artery territories) for segmental wall assessment with convention and advanced echocardiography as well as myocardial contrast perfusion echocardiography

Parameter			Feasibility Patients *n* (%)	Feasibility *n*/total segments (%)	Value	No coronary artery disease	Significant coronary artery disease	*p* value
2D segmental thickening assessment (wall motion score index)		LAD	36 (90%)	329/360 (91%)	2 (1.5–2.5)	2 (1.4–2.6)	1.9 (1.5–2.6)	0.75
		LCx	34 (85%)	142/160 (89%)	2 (1–2)	2 (1–2)	2 (1–2.5)	0.75
		RCA	40 (100%)	233/240 (97%)	2 (1–2)	2 (1–2)	2 (1–2.1)	0.98
Longitudinal strain analysis by speckle tracking echocardiography (%)		LAD	25 (63%)	263/360 (73%)	− 6.25% (− 10 to – 2)	− 5.4% (− 10 to − 1)	− 7% (− 18 to − 5)	0.29
		LCx	34 (85%)	137/160 (86%)	− 8.1% (6)	− 7.9% (6)	− 9.3 (5)	0.59
		RCA	36 (90%)	210/240 (88%)	− 9.2% (5)	−9% (− 11 to − 6)	− 7.5% (− 16 to − 7)	0.9
Myocardial contrast perfusion echocardiography	Subjective assessment (*n*)	Overall	–	400/640 (62.5%)	4	1	3	*0.01*
		LAD	21 (53%)	260/360 (72%)	4	1	3	*0.01*
		LCx	8 (20%)	58/160 (36%)	2	0	2	*0.02*
		RCA	21 (53%)	142/240 (59%)	2	0	2	*0.02*
	Quantitative assessment (dB/s)	Overall	–	423/640 (66%)	3.2 (3–4)	3.3 (3–4)	2.6 (1–3)	*0.03*
		LAD	26 (65%)	268/360 (74%)	3.3 (3–4)	3.3 (3–4)	2.5 (1.4–3)	*0.04*
		LCx	13 (33%)	72/160 (45%)	2.9 (1)	3.1 (3–4)	1.6 (1–3)	*0.02*
		RCA	23 (58%)	154/240 (64%)	3.0 (1)	3.1 (1)	2.5 (1)	0.28

*LAD* left anterior descending coronary artery, *LCx* left circumflex coronary artery, *RCA* right coronary artery

The italicized data was simply meant to highlight the values which are statistically significant (ie: have p values <0.05)

### Detection of significant coronary artery disease

**Fig. 3:**
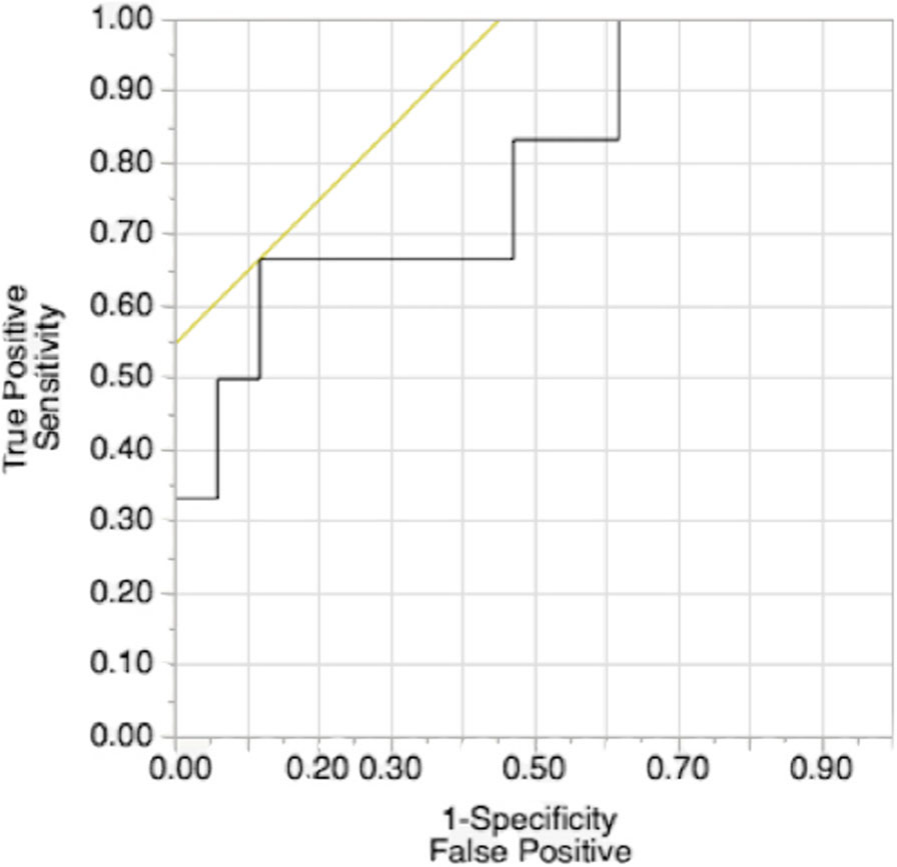
Receiver operating curve for myocardial contrast perfusion echocardiography (MCPE) for determining presence vs absence of significant coronary artery disease (value of 2.9 dB/s had 67% sensitivity and 88% specificity)

Based on ROC curve analysis, the optimal MCPE cut-off value for the presence vs absence of significant CAD is 2.9 dB/s (see Fig. 3) which had 67% sensitivity and 88% specificity. We found a positive predictive value of 50% and a negative predictive value of 91%. Of the 3 patients who were found to have perfusion deficits on MCPE, 2 had angiography and the other an MRI. Association between clinical acumen and quantitative MCPE or subjective MCPE analysis in predicting significant CAD is shown in Table 3. Clinical acumen showed no significant association in predicting presence of significant CAD (OR 0.64, *p* = 0.091); however, if quantitative or subjective MCPE analysis was included in the model, incremental improvement and significant association was seen (OR 17.15, *p* = 0.013; OR 23.05, *p* = 0.010 respectively). The best association was seen with subjective MCPE analysis alone (OR 33.0, *p* = 0.003).

**Table 3:** Logistic regression analysis of association between clinical acumen, quantitative, or subjective myocardial contrast perfusion echocardiography (MCPE) analysis in predicting presence of significant coronary artery disease

Model	Covariates	Odds ratio [confidence intervals]	*p* value	*R* ^2^
1	Clinical acumen	0.64 [0.37–1.10]	0.091	0.09
2	Quantitative MCPE analysis	10.3 [1.41–75.7]	0.022	0.15
3	Subjective MCPE analysis	33.0 [2.57–424.0]	0.003	0.26
4	Clinical acumen	0.57 [1.03–1.75]	0.049	0.27
	Quantitative MCPE analysis	17.15 [1.61–183.1]	0.013	
5	Clinical acumen	0.74 [0.38–1.40]	0.352	0.28
	Subjective MCPE analysis	23.05 [1.69–313.6]	0.010	

*MCPE* myocardial contrast perfusion echocardiography

Inter-rater variability was assessed by M.S. vs S.O. in random 6 patients (15%). Inter-rater variability was reasonable with mean absolute difference in estimated blood flow (± SD) of 0.31 dB/s (± 1.5) and expressed as percentage of the mean of 9% (± 36).

## Discussion

We found performing myocardial contrast perfusion echocardiography (MCPE) to be feasible in critically ill patients, with no significant adverse reactions seen. Importantly, clinical acumen alone (using clinical history, ECG, echo and Troponin I levels) did not have significant association with predicting the presence of significant CAD. However, if quantitative or subjective MCPE analysis was included in the assessment, then significant association was seen.

Troponin I levels are frequently elevated in the ICU population; however, only a minority of patients have significant CAD and/or acute coronary artery occlusion (e.g. from thrombus or acute antherosclerotic plaque rupture). Ko et al. found only 30% of ICU patients with a clinical diagnosis of myocardial infarction ended up with a diagnosis of significant CAD on coronary angiography [1]. Our study findings support this sentiment with neither conventional segmental myocardial thickening analysis on 2D echo images nor longitudinal strain analysis by STE able to distinguish significant CAD presence either. This is mirrored in the fact that both specialists in ICU and cardiology were unable to reliably predict significant CAD based on convention means. The diagnosis of significant CAD can be extremely difficult in the critically ill, and this is potentially dangerous given the risk of sending a patient for angiography (including radiographic contrast administration) or prophylactic use of anti-platelet and anticoagulation agents. Our study indicates that MCPE use could aid in helping make the correct choice in whom to send for angiography.

Wei et al. pioneered the method of using echo contrast to estimate myocardial blood flow, and it appeared to be correlated well with radiolabeled microsphere myocardial blood flow [17]. This technique has been used in the cardiology sphere for decades, and large safety studies have been performed on the use of contrast [18]; however, to our knowledge, there is little information on the utility of MCPE in the critically ill. A difficulty we faced, particularly in the critically ill, is the confidence in MCPE being able to differentiate acute coronary artery occlusion (e.g. type 1 myocardial infarction) vs chronic, flow-limiting, significant CAD (a form of type 2 myocardial infarction). The distinction may be important as it impacts the risk-benefit analysis of having urgent angiography performed. The strength of MCPE use in the ICU patient may therefore be in determining those with significant CAD vs those without. In this regard, we see the MCPE analysis technique not to replace angiography or other imaging modalities, but to help reduce some of the variation seen in clinical assessment with conventional means and may help direct decision making. It is far from perfect but may be a useful addition to the critical care physician armamentarium.

We found MCPE much better at assessing the apical than the basal segments, which may mean that this method may be more efficient at assessing left anterior descending coronary artery territory ischaemia than the other coronary arteries. Indeed, feasibility was much better in the LAD territory than the LCx or RCA. This is disappointing given that posterior ischaemia may be more difficult to diagnose in critically ill patients; however, other studies have reported similar results [19].

### Limitations

There are several limitations to our small, single-centre, observational study. The primary issue is the lack of a recognized reference standard to exclude acute coronary artery occlusion (angiography or cardiac MRI) in a significant portion of our patients (e.g. 28% of our patients had a simple normal follow-up echo). However, often, once an acute event had settled, the risk of angiography may outweigh the benefit (e.g. likely stress-induced cardiomyopathy in a patient with subarachnoid haemorrhage). Those with an initial abnormal echo were not excluded and then normalized findings at a later date still had coronary artery disease similar to those having exercise stress tests; however, we did find a high specificity and negative predictive value in those with significant CAD found. In addition, the analysis was performed by a single operator (S.O.) who was at times the treating clinician. The blinding of the analysis was therefore, at times, not possible. Patients were not consecutive as S.O. was the sole MCPE operator in the unit and patient inclusion was based on other clinicians highlighting potential subjects, during work hours. Several patients are likely to have been missed and this suggests selection bias. However, this was primarily a feasibility study and information may be useful as pilot data to guide future studies. The analysis of our data into coronary artery territories may also be inaccurate as individual patient coronary artery blood flow does not always follow the standard anatomical boundaries.

### Future research

The use of MCPE in determining large defects in myocardial blood flow may be useful in the ICU environment. Further research should be done in this area to try and help improve our early recognition of significant CAD. Studies should focus on true blinded assessment, multi-operator analysis, and objective MCPE analysis being performed immediately after the study has taken place. Additional studies may assess the significant variation seen in clinicians estimating the likelihood of significant CAD being present and if MCPE analysis could help reduce some of this variation.

## Conclusion

We found MCPE estimation of myocardial blood flow to be feasible in critically ill patients and found no adverse events. Clinical correlation alone is extremely variable and unable to reliably determine the presence or absence of significant CAD, yet we know that unnecessary intervention or treatment can be harmful. MCPE may be able to improve our recognition in predicting significant CAD in the critically ill.

## Abbreviations

CAD: Coronary artery disease
Echo: Echocardiography
LAD: Left anterior descending coronary artery
LCx: Left circumflex coronary artery
LV: Left ventricle
MCPE: Myocardial contrast perfusion echocardiography
RCA: Right coronary artery
ROI: Region of interest
SI: Signal intensity
